# Structure-based engineering of papillomavirus major capsid L1: controlling particle assembly

**DOI:** 10.1186/1743-422X-4-3

**Published:** 2007-01-08

**Authors:** Brooke Bishop, Jhimli Dasgupta, Xiaojiang S Chen

**Affiliations:** 1 Molecular and Computational Biology, University of Southern California, Los Angeles, CA 90089, USA; 2 Biochemistry and Molecular Genetics, University of Colorado HSC, Denver, CO 80262, USA

## Abstract

The outer shell of the papillomavirus particle is comprised of 72 pentamers of the major capsid L1 protein arranged on a T = 7 icosahedral lattice. The recombinant L1 can form T = 7 virus-like particles *in vitro*. The crystal structure of a T = 7 papilloma virion has not yet been determined; however, the crystal structure of a T = 1 particle containing 12 pentamers is known. The T = 1 structure reveals that helix-helix interactions, through three helices–h2, h3, and h4–near the C-terminus of L1, mediate the inter-pentameric bonding that is responsible for T = 1 assembly. Based on the T = 1 crystal structure, we have generated a set of internal deletions to test the role of the three C-terminal helices in T = 7 assembly. We have demonstrated that the h2, h3, and h4 near the C-terminal end of L1 are important for the L1 structure and particle assembly. In particular, we found that h2 and h3 are essential for L1 folding and pentamer formation, whereas h4 is indispensable for the assembly of not only T1, but also of the T7 virus-like particle.

## Background

Papillomaviruses are non-enveloped DNA tumor viruses. Infection by papillomaviruses is associated with tumorigenesis in experimental animals and in humans (reviewed in [5]). The virus particle is comprised of 72 pentamers of the major capsid protein (L1) on the outer surface, arranged on a T = 7 icosahedral lattice (reviewed in [3,5]). A minor capsid, L2, is located internal to the L1 shell. The viral genomic DNA is packaged within the L1/L2 capsid as a minichromosome (reviewed in [5]).

The recombinant L1 capsid alone can assemble into the virus-like particle structure *in vivo *and *in vitro *[4,6,7,10-12,15]. Thus, the L1 protein contains all the information needed for the particle assembly. The *in vitro *assembly of L1 occurs spontaneously under high salt or low pH conditions [1,2]. The sizes of particle assembly can be regulated by N-terminal truncations. For example, HPV16 L1 lacking the first 10 residues assemble into T = 1 particle, but L1 deletions missing nine or fewer residues from the N-terminus assemble into T = 7 particles [1,2].

**Figure 1:**
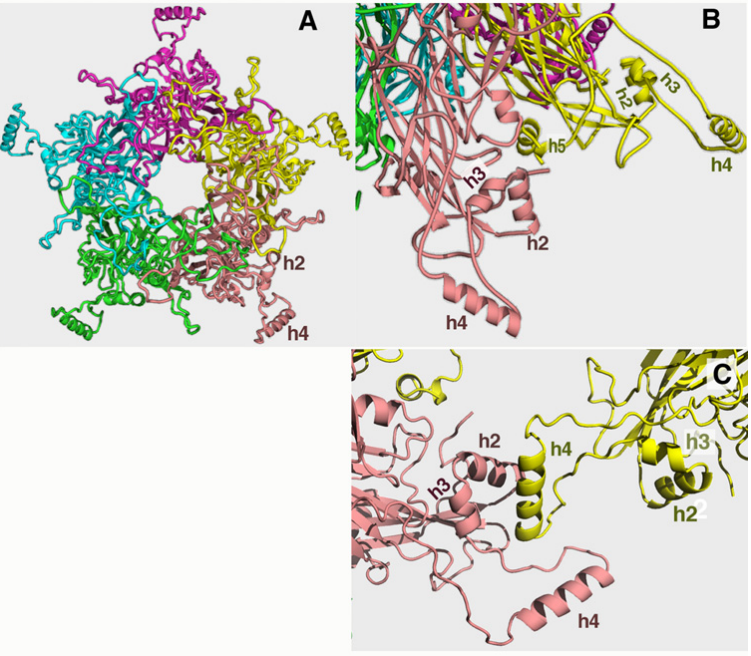
Structural elements of L1 important for papillomavirus particle assembly. **A**) The top view of an L1 pentamer, showing the lateral projections from each of the five monomers (each in a discreet color). **B**) A close-up view of the lateral projections two monomers, showing the detailed positions of h2, h3, h4, and h5 at the C-terminus of L1. **C**) The inter-pentameric helix-helix interactions between two pentamers in the T = 1 particle. The h4 of one pentamer (in yellow) interacts with h2 and h3 of another pentamer (in pink).

The atomic structure of a T = 7 papilloma virion has not yet been determined. However, the crystal structure of a smaller particle structure, a T = 1 particle containing 12 pentamers, has been solved [2]. The high resolution T = 1 structure reveals the L1 pentamer structure and the pentamer-pentamer interactions essential for the assembly of the T = 1 particle. The L1 pentamer has five elbow-like lateral projections that are composed of the approximately 100 residues at the C-terminus (Fig. 1A). Each projected elbow consists of an α-helix (helix 4 or h4) anchored to the core structure through two other helices, h2 and h3 (Fig. 1B). Pentamers are linked through strong hydrophobic interactions between h4 on one pentamer with h2 and h3 of a neighbor (Fig. 1C). The remaining C-terminal residues return to the L1 core to form a fifth helix, h5 (Fig. 1B).

By fitting the L1 atomic structure of from the T = 1 particle into the cryo-EM reconstruction of BPV [14], an atomic model for T = 7 assembly was proposed [9]. In the fitted T = 7 model, the laterally projected C-terminal fold of L1 seen in the T = 1 structure is rearranged to extend into the neighboring pentamers. This C-terminal arm exchange between the neighboring pentamers is similar to the "invading arm" model of the polyomavirus VP1 T = 7 virion structure [8,13]. However, the roles of the helix-helix interactions observed in the T = 1 structure are not defined in the T = 7 assembly model. In order to understand the functional roles of h2, h3, and h4 in T = 7 assembly, we have made structure-guided internal deletions of these helices on the L1 of two different HPV types. We have demonstrated that h2 and h3 are essential for L1 folding and the production of the pentameric L1 and that h4 is indispensable for the assembly of T = 7 particles.

## Results

### Internal deletions of HPV16 L1

**Figure 2:**
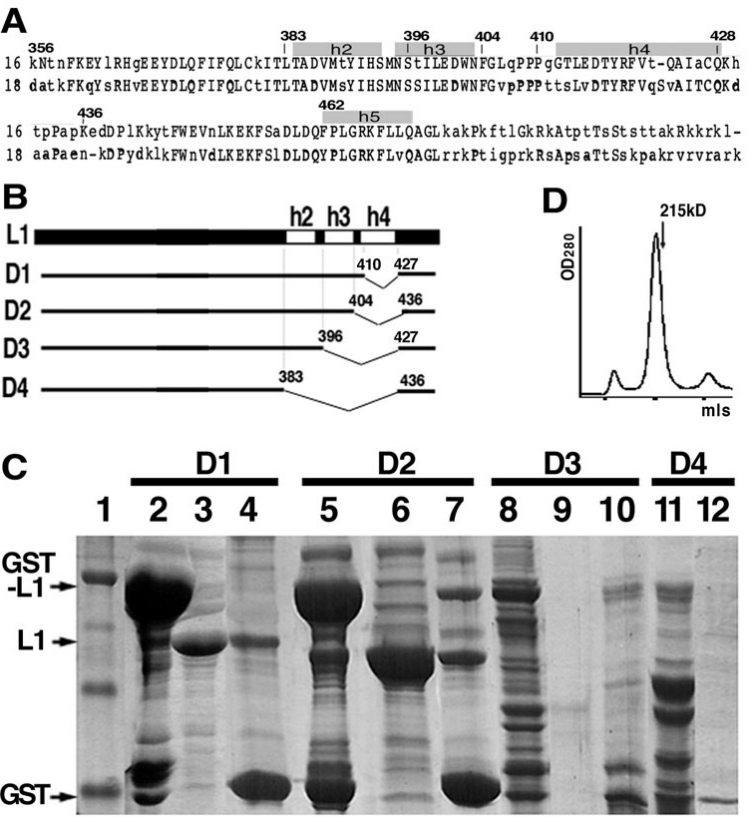
Structure-based internal deletions of HPV16 L1. **A**) Sequence alignment of HPV 16 and HPV18 L1, showing the conserved regions of h2, h3, and h4. **B**) Diagram showing the design of the four internal deletions of HPV16 L1. D1 has a deletion within h4 and D2 removes h4 and a few residues outside the boundaries of h4 on both ends. **C**) Solubility and stability of the four constructs of HPV16 L1. Lanes 2–4: D1-L1 mutant; lanes 5–7: D2-L1 mutant; lanes 8–10: D3-L1 mutant; and lanes 11–12: D4-L1 mutant. Lanes 2, 5, 8, and 11 show the proteins eluted from the resin before thrombin digestion; lanes 3, 6, and 9 show the proteins eluted from the column after thrombin digestion; and lanes 4, 7, 10, and 12 show the proteins in the resin after thrombin digestion and elution. **D**) Size-exclusion chromatography analysis of the D1-L1 eluted from the column after thrombin treatment, showing the typical elution profile on Superdex-200. The major peak has an apparent molecular weight of ~235 kD, the expected size for a pentameric L1.

The crystal structure of the T1 particle of HPV16 L1 capsid protein shows 60 L1 molecules assembled through pentamerization interactions and inter-pentameric bonding [2]. The L1 protein with β-barrel core oligomerizes into pentamers (Fig. 1A). The pentamers further associate with each other to assemble into icosahedral particles of different T numbers. In the T = 1 crystal structure, the pentamer-pentamer interactions are formed through interactions of three helices near the C-terminus, h2, h3, and h4 (Fig. 1B, C). However, it is not clear if these helices are important for the T = 7 virion particle assembly. To determine the role of h2, h3, and h4 in the HPV16 papillomavirus particle assembly, four internal deletions, D1, D2, D3, and D4, were constructed (Fig. 2A, 2B). Briefly, in the D1 and D2 constructs h4 was deleted, in D3 both h4 and h3 were deleted, and in D4 all three helices were deleted. Because the full-length L1 can be expressed and purified successfully as GST-L1 fusion [1], the deletion mutants were constructed to express GST-L1 proteins.

### Solubility and stability of the deletion mutants of HPV16 L1

Because the L1 expression level and solubility could not be easily detected by directly analyzing the cell lysates by SDS-PAGE, we performed partial purification of the GST-fusion proteins from the cell lysates using affinity chromatography. The GST-L1 protein from the supernatant fraction of the lysates was passed through a glutathione resin column. After washing the column with 10× bed volumes of buffer L (25 mM Tris-Cl, pH 8.0, 0.25 M NaCl, 1 mM EDTA, 2 mM DTT), the resin was incubated with thrombin to cleave L1 from the GST fusion.

The proteins bound to the resins before and after thrombin treatment, as well as those in the eluate after thrombin treatment, were analyzed by SDS-PAGE (Fig. 2C). D1 and D2 constructs produced large quantities of soluble GST-L1 fusion proteins (lanes 2 and 5, respectively). D3 produced some GST-D3-L1 fusion (lane 8 in Fig. 2C), but many degradation bands were observed in the fraction before thrombin treatment. These are presumably L1 degradation products that are still fused with GST at the N-terminus of L1. There was almost no detectable full-length GST-D4-L1 fusion (lane 11 in Fig. 2B), indicating that this protein was even more severely degraded than the GST-D3-L1 protein (compare lanes 8 and 11).

After thrombin digestion of the GST-L1 fusions on the glutathione resin, soluble D1- and D2-L1 were eluted from the column (lanes 3 and 6, respectively). For D3-L1, however, no intact protein was present in the elution after thrombin cleavage; D3-L1 may be degraded by thrombin due to an unfolded or misfolded conformation.

The L1 proteins eluted from the glutathione column were analyzed using size-exclusion chromatography on Superdex-200 (Fig. 2D). A single major peak with the elution position expected for a pentamer was present for both D1- and D2-L1. These results demonstrated that h4 deletion mutants D1-L1 and D2-L1 were soluble and stable and that they fold and oligomerize into pentamers. However, when the deletion extended to h3 (in D3) or to h2 (in D4), the proteins were degraded into multiple fragments, before and during thrombin treatment.

### D2 mutants of another HPV type, HPV18 L1

**Figure 3:**
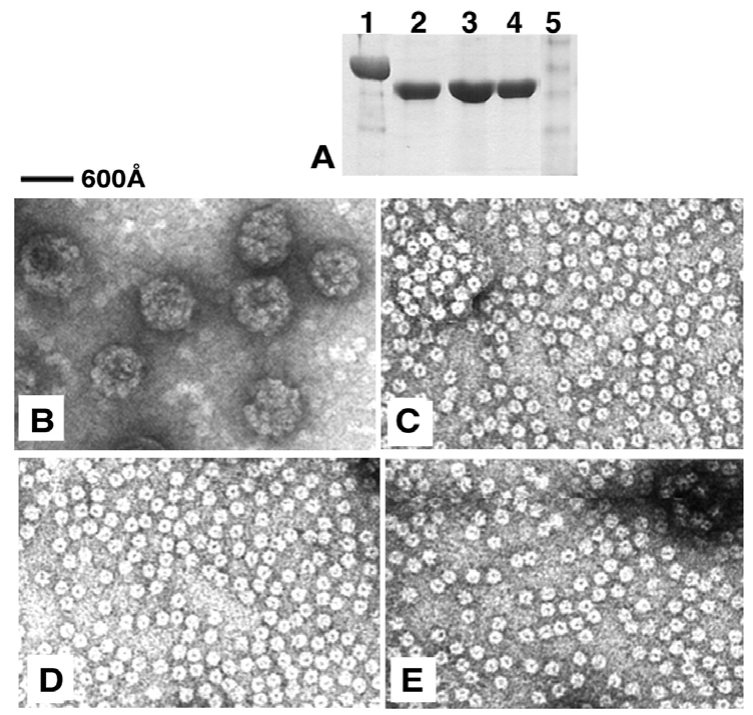
EM examination of particle assembly of L1 mutant proteins. **A**) The purified proteins from four different L1 constructs. Lane 1: full-length HPV16 L1; lane 2: D1-L1 deletion mutant of HPV16 L1; lane 3: D2-L1 deletion mutant of HPV16 L1; lane 4: D2-L1 deletion mutant of HPV18 L1. **B-E**) EM images of the purified proteins treated under assembly condition. The proteins were in incubated in the assembly buffer at 25°C for 30 minutes. Uranyl acetate was then used to treat the protein samples on a carbon grid for EM examination. The scale of the images is indicated by the bar above panel B. The images shown are: (B) full-length HPV16 L1; (C): D1-L1 mutant of HPV16 L1; (D): D2-L1 mutant of HPV16 L1; (E): D2-L1 mutant of HPV18 L1.

Since both D1 and D2 deletion constructs of HPV16 produced soluble L1 proteins, we attempted the equivalent of a D2 deletion on HPV18 L1 (Fig. 2A, 2B). As expected, the D2 deletion mutant of HPV18 was expressed and purified using the same protocol for HPV16 L1, and the protein behaved like a pentamer in size-exclusion chromatography in a buffer containing 25 mM Tris-Cl, pH 8.0, 0.2 M NaCl (data not shown). All the purified deletion mutants of the L1 proteins can be concentrated to approximately 20 mg/mL (Fig. 3A). The yield of the purified L1 proteins for D1 and D2 deletion constructs of HPV16 and HPV18 were roughly comparable, varying in the range between 0.7 mg to 1.3 mg per litters of cell culture.

### Particle assembly assays of D1 mutants of L1

**Figure 4:**
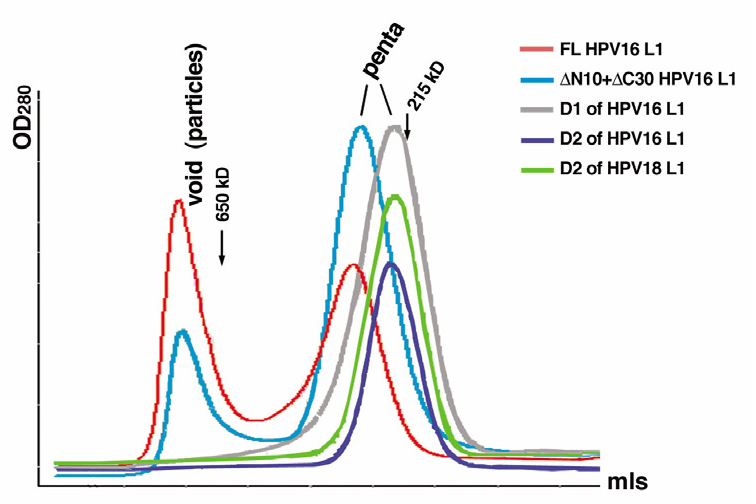
Examination of particle assembly of L1 mutants by size-exclusion chromatography. The proteins were incubated in assembly buffer at 25°C for 30 minutes before analysis on the Superdex-200 column (16/60, Pharmacia) at a flow rate at 1 mL/min. The positions of the molecular standards are indicated by arrows. An HPV16 L1 construct, ΔN10+ΔC30, shown previously to assemble into T = 1 particles, was included as a control (blue line).

We used two methods, electron microscopy (EM) and size-exclusion chromatography, to examine whether the purified proteins from the deletion constructs of HPV16 and 18 assembled into particles. The L1 proteins (at a concentration of ~0.1 mg/mL) were first incubated in a buffer known to promote icosahedral particle assembly [1] for 30 minutes at room temperature. The proteins were then examined using EM and size-exclusion column chromatography on Superdex-200. As shown in Fig. 3C–E, after incubation in assembly buffer, only pentamers, and no viral particles, were detected by EM examination of the D1-L1 and D2-L1 deletions of HPV16 and D2-L1 of HPV18. Under the same conditions, T = 7 particles, as well as some free pentamers, were observed for the full-length HPV16 L1 (Fig. 3B). Because of the potentially disruptive acidic conditions in the negative staining for EM examination, the particle assemble reaction was also examined by size-exclusion chromatography. The chromatography result showed that each of the three L1 mutants eluted as a single pentamer peak after assembly treatment (Fig. 4). In contrast, the full length HPV16 L1 showed an additional peak at void volume after assembly treatment, consistent with T7 particle assembly observed using EM.

## Discussion

The T = 1 particle structure shows that h2, h3, and h4 near the C-terminal end of L1 are the solely responsible for making inter-pentameric contacts required for the particle assembly [2]. There are the obvious differences between the T1 structure and the proposed assembly model of T7 [9], and the roles of h2, h3, and h4 in T7 assembly are not defined. We have selectively deleted each of the three helices from papillomavirus L1. The biochemical and structural characterization of the deletion mutant proteins demonstrated the important roles of these C-terminal helices in the folding and assembly of L1 capsid.

The deletion mutants of HPV16 L1 lacking h4, h3, or h2 were expressed as GST-fusions in *E. coli*. The mutant proteins with partial or entire h4 deletion, D1-L1 and D2-L2, were similar to the full-length wild-type L1 in protein expression and behavior by size-exclusion chromatography. Like wild type, D1-L1 and D2-L2 were expressed at quite high levels, and a good fraction of each expressed proteins was soluble and could be purified as pentamers (Fig. 2C). This result demonstrated that h4 is not required for L1 folding and pentamerization.

In contrast, when h3 and h2 were deleted and fusion proteins (GST-D3-L1 and GST-D4-L1) were expressed, little intact GST-L1 fusion protein was detected (Fig. 2C). Instead, multiple smaller protein bands were present in the SDS-PAGE gels. Because only a GST-fusion can bind the glutathione resin of the column used for purification, the smaller protein bands must contain intact GST plus L1 fragments of various lengths due to C-terminal degradation. Thrombin treatment of these GST-fusions did not produce discreet L1 fragments, indicating that both D3-L1 and D4-L1 were unfolded and sensitive to protease degradation. Consistent with this severe degradation of these deletion mutants of L1 is that there are six potential thrombin cleavage sites within the HPV16 L1 sequence and many Arg and Lys residues can serve as cleavage sites for other proteases. Thus, we conclude that h2 and h3 are important for the folding and integrity of HPV L1 structure.

Interestingly, a small helix at the C-terminus, h5 (Fig. 1B), was also shown to be critical for L1 folding [1,2]. A common feature of h2, h3, and h5 in the x-ray structure [2] is that all helices have a highly hydrophobic side that packs with the core structure of L1. Disrupting these hydrophobic packing interactions will obviously affect the folding of L1.

The deletion of h4 in another papillomavirus, HPV18, resulted in a mutant that behaved like the HPV16 D2-L1 mutant. This result confirmed that the L1 structure is conserved among different HPV types, which is not surprising considering the amino acid sequences of L1 proteins are highly conserved. We predict that the same D2 deletion of any other papillomavirus L1 should have the same properties.

When the D1-L1 and D2-L2 proteins of HPV16 and 18 were incubated under assembly conditions, pentamer structures, but no T = 7 particles, were visualized by EM examination (Fig. 3C–E). If the deletion mutants assemble into particles, these particles might be unstable to the acidic conditions used during EM sample preparation, which requires uranyl acetate treatment. To rule out this possibility, size-exclusion column chromatography was used to analyze the deletion constructs after assembly treatment (Fig. 4). The size-exclusion analysis showed that, in contrast to the full length L1, the D1-L1 and D2-L2 mutants lacking h4 failed to form assemblies larger than the pentamers, confirming the results from EM study.

In conclusion, we have demonstrated that the structural elements h2, h3, and h4 near the C-terminal end of L1 are important for the assembly of papillomaviruses into particles. The h2 and h3 regions are essential for L1 folding and pentamer formation, whereas the h4 region is indispensable for the assembly of not only T1, but also of the T7 virus-like particle.

## Materials and methods

### Cloning and deletion

The full-length L1 clones of HPV16 and HPV18 in pGEX-2T were used as parent clones for generating the deletion mutants of D1, D2, D3, and D4 as shown in Fig. 2A, 2B. The L1 proteins were cloned downstream of GST to express as GST-L1 fusions in this *E. coli *expression vector. The standard molecular cloning methods, including PCR amplification, enzyme digestion, DNA end ligation, and transformation, was used to obtain the deletion mutants of L1 as shown in Fig. 2A, 2B. All deletion clones were confirmed by sequencing the whole DNA insert to ensure the correct deletion and the wilt type sequences for this study.

### Protein purification

The protein expression and purification of all L1 deletion mutants was carried out essentially as described preciously [1,2]. Briefly, 0.2 mM IPTG was use to induce protein expression overnight at room temperature (RT). After cell lysis by sonication in buffer L (50 mM Tris-HCl, pH 8.0, 0.2 M NaCl, 1 mM DTT, 1 mM EDTA, 10 mM PMSF), urea (ultrapure grade) was slowly added to the lysate to a final concentration of 3.0 M. The mixture was incubated at RT for one hour with gentle shaking, and then dialyzed against three changes of buffer over an 18 hour period. After centrifugation at 25,000 × g for 75 min, the supernatant was passed through a glutathione affinity column to bind GST-L1 fusions. Usually, 10 ml glutathione resin was used for supernatant from 12 liter cell culture. The column was washed with 10× bed volumes of buffer L to wash away contaminating proteins. At this point, an additional washing step using 10× bed volumes of 2.3 M urea can clean up the small amount of the contaminated groEL. L1 was then cleaved from the GST fusion using thrombin (Sigma, T6634) in an approximate ratio of 100 μg GST-L1 to 1 NIH unit of enzyme. The digestion was carried out at 4°C overnight. L1 was further purified by Superdex-200 (60/16 column) size-exclusion chromatography. During the purification process, keep all the reagents at 4°C whenever possible.

### Analysis of *in vitro *particle assembly by EM and size-exclusion chromatography

Wild-type L1 can assemble into particles under known *in vitro *assembly conditions [1,2]. The assembly reaction was performed by incubating purified L1 protein in assembly buffer (1 M NaCl, 40 mM sodium acetate, pH 5.4) at 25°C for 30 minutes, at a protein concentration of approximately 0.1 mg/mL. For EM analysis, samples were adsorbed to glow-discharged, carbon-coated copper grids (EM Science) and negatively stained with 2% uranyl acetate. Grids were examined in a JEOL 1200 transmission electron microscope at 80 kV and 60000× magnification. For size-exclusion chromatography analysis, samples were injected directly into the sample loop and onto a Superdex-200 column (16/60, Pharmacia) at a flow rate of 1 mL/minute in the assembly buffer. The protein peaks were detected by an UV monitor at a wavelength of 280 nM.

## Competing interests

The author(s) declare that they have no competing interests.

## Authors' contributions

BB and JD participated in making some of the constructs and performed the experiments. BB and XC designed the experiments.
